# Supplementary material for: Development of a conditional plasmid for gene deletion in non-model Fusobacterium nucleatum strains
Source: Appl Environ Microbiol. 2025 Jan 24;91(2):e01816-24. doi: 10.1128/aem.01816-24 (PMC11837546; doi:10.1128/aem.01816-24)
Supplement: Fig. S1 and S2 — Fig. S1, qRT-PCR analysis; Fig. S2, alignment of RadD homologs. [file aem.01816-24-s0001.docx]

**Development of a Conditional Plasmid for Gene Deletion in Non-Model *Fusobacterium nucleatum* strains**

**Peng Zhou, Bibek G C and Chenggang Wu^†^**

*Department of Microbiology & Molecular Genetics, the University of Texas Health Science Center, Houston, TX, USA*

**^†^** To whom correspondence should be addressed. Tel. (+1) 713 500 5437; E-mail: [chenggang.wu@uth.tmc.edu](mailto:chenggang.wu@uth.tmc.edu)

Running Title: Gene deletion in non-model *F. nucleatum strains*

Keywords: *Fusobacterium nucleatum*, *repA*, theophylline-responsible riboswitch, gene deletion

**Fig. S1**: The relative expression of the *radD* transcript was analyzed by qRT-PCR in *Fusobacterium nucleatum* strains ATCC 23726, CTI-2, and 21_1A. Cultures of each strain were grown to an OD_600_ of 1.0 in a TSPC medium, followed by RNA extraction. *radD* transcript levels were quantified using qRT-PCR, normalized to the reference gene *gyrB*, and expressed relative to strain ATCC 23726, assigned an arbitrary value of 1. Data are presented as the mean ± SEM from three independent biological replicates.


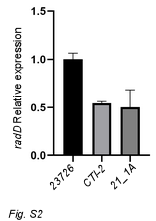


**Fig. S2: Alignment of RadD Homologs.** The region highlighted in yellow (amino acids 44-200) was selected for antibody generation. Variations in amino acid sequences among the homologs are indicated in red.

23726RadD MKDYNKVESCLKSFLKNNKRLSYSMALLITFLINGGFSYADEAVQVPLRTEIKTRIEKEQ

21_1ARadD MKDYNKVESCLKSFLKNNKRLSYSMALLISFLINGGFSYADEAIQVPLRTEIKTRIEKEQ

CTI2RadD MKDYNKVESCLKSFLKNNKRLSYSMALLITFLINGGFSYADEAVQVPLRTEIKTRIEKEQ

10953RadD MKDYNKVESCLKSFLKNNKRLSYSMALLISFLINGGFSYADEAIQVPLRTEIKTRIEKEQ

*****************************:*************:****************

23726RadD ENISQMLKEADESMKDIELKIKKLTQRGEFWVKPLEKSYQGFIFANWGNYSKNKNKTESN

21_1ARadD ENISQMLKEADESMKDIELKIKKLTQRGEFWVKPLEKSYQGFIFANWGNYSKNKNKTESN

CTI2RadD ENISQMLKEADETMKDIELKIKKLTQRGEFWVKPLEKSYQGFIFANWGNYSKNKNKTESN

10953RadD ENISQMLKEADKSMKDIELKIKKLTQRGEFWVKPLEKSYQGFIFANWGNYSKNKNKTESN

***********::***********************************************

23726RadD FNGPEYSASYGRNMGYGQFSNGKYYGEYGIVKNPLEFVDKIDFGANITPKAVIEKTIIEK

21_1ARadD FNGPEYSASYGKNMGYGQFSNGKYYGEYGIVKNPLEFVDKIDFGANITPKAVTEKTIVEK

CTI2RadD FNGPEYSASYGRNMGYGQFSNGKYYGEYGIVKNPLEFVDKIDFGANITPKAVIEKTIVEK

10953RadD FNGPEYSASYGSSMGYGQFSNGKYYGEYGIVKNPLEFVDKIDFGANITPKAVTEKTIVEK

*********** .*************************************** ****:**

23726RadD TVIKKDITAPSVTPPTVEVGEITLTAPEEVAIDEMTPPNEPNPSVTTPSTVPALQGITVA

21_1ARadD TVIKKDITAPSVTPPTVEVGEITLTAPEEVAIVEMTPPNEPNPSVTTPSTVPALQGITVA

CTI2RadD TVIKKDITAPSVTPPTVEVGEITLTAPEEVAIGEMTPPNEPTVNVQIPGTIPTLSPISVT

10953RadD TVIQKIIEAPRVTPPIVTIATITPPTVSTPTIGTMSEPGAITVNVTAPGTPPTLSAITVS

***:* * ** **** * :. ** .: . :* *: *. . .* *.* *:*. *:*:

23726RadD AVSEVNVTPSTPEVAAAPTINAPGVQPPATPAGFTPRLITPPEAPEDIVITPPGEVQANL

21_1ARadD AVSEVNVTPSTPEVAAAPTINAPTVTPPATPAGFTPRLITPPEVPADIVVTPPTITPPTL

CTI2RadD PVTALNINPTPPTVGDAPTVTAPTVTPPATPAGFTPRLVSPPTVAEKTVNISPVPNPPST

10953RadD AVPEVSITPQPPEVAEAPVVTEPTVTAPATPAGFTPRLVSPPTVAEKIVNISPVPNPPST

.*. :.:.* .* *. **.:. * * .***********::** .. . * .* ..

23726RadD TGSGAN-PTTYYYWWNG--------NKGAISQISLTAGTFNIESGNTINVNG--------

21_1ARadD LGGGANPQADRYYYWDG--------NDGAISQVNVTSGTISITGNVTNYSNGNMDLTLNN

CTI2RadD DVAYQAVPANVTGYRNPGDKI----NGGLMSQLELTAGTFNIYDMVG----------TSG

10953RadD DVHYQDVPNNSTGWYGGTGSSQVTGNNGLISQLDLTAGNYEMYFRGHNKGQDHKFSNAKD

: . * * :**:.:*:*. .:

23726RadD YQAKAFTGTTPQGTKPSDGDYVINQQFFHTLLNVAYSEYSNGVVINNNRAG---------

21_1ARadD FHVSAYPGTSPSGTAPTSGTYKLNRRFFNTLLNVPYSEFSSGVTINYNYKIGGSNPQNNP

CTI2RadD FKYSFTGATATNGNSPSSGYASLPSNDTGTLNGEAFYRHGG-------------------

10953RadD SAGILPSTYSVDGMNKVAFYGIGGKPLVTIPN----------------------------

: .*

23726RadD -----FTLINLETEGQVSGNLDNRVGT-YIDSAKRDKLRSYQMYSGIKGQDN--TELLFI

21_1ARadD PTPYEGAVINLETEGNVAGNLSTAVTKNQITESKKNILKGYQSYSGITGNDNGATELLFI

CTI2RadD --------KALTTIGSAVTINAVGKNNGAPLNSIFYLGNNTTNPNSESKLINKATVNLYG

10953RadD ---------------TVTIKAVGNNTTNSPLNAIFYLGNNTTNPNSESKLINKATVNLYG

. . .: .. .. . * * *:

23726RadD NKGTVNLNADNTTYLFTTSHNDGNLRTNYLDNEGTISAKG--KNSIIIKHSPDTNQAKAW

21_1ARadD NKGNINLISEKGIYIFTTTHTGGNNRTNYLDNEGTISAKG--KESVIIKHTPDTAEGRGW

CTI2RadD NKIAVSNIDNVSSAGDITFVNEGNIIGHAVSEFGGYQGNS--NVGNYIFGGYSYGNAGVD

10953RadD NKIAVSNIDNVSSSGNITFVNEGNIIGHAESGEYASGKVGNSAVGNFIFGGYSYGDAGVD

** :. : * . ** : . . . * . :.

23726RadD IYSNN--GIMKAEGKGSIVYGAAYSYLGSGRAAFVNDGTIEVSGEEAIGVILPRKTG--N

21_1ARadD IYSNSSSGKMYADGEGSVIMGWAYKHLQHGRAAFVNAGEIKVRGKRAVGIFMADDTNPNN

CTI2RadD KIENGASGKVIFYAPNSVGWAYTSGATQTVKRSSINNGTMKLYGHHSLGIATDNDATVEQ

10953RadD TIENGVGGNVTFYAPNSVGWAYTSSSTQAVKRSSINNGIMKLYGHHSLGIATDGDASVEQ

.*. * : . .*: . : : : :* * ::: *..::*: .: :

23726RadD SELANGHLVWLKKPISLKGKKSMGFVAQNTNISNEKN--LVKFNIDAEKAIGILQDVEG-

21_1ARadD TTMAAGSSVYLTKPIDLLGDRSMGWVSQNTGVSAGNGGYFVQFNIGNEVQDATLGESEGD

CTI2RadD MSWAD---IQLNTPIEILGDQSVGASIKTE-PDETKSTNFFGSKWNIKIGGLNGSSQDAT

10953RadD MSWAD---IQLNTPIEILGDQSVGASIKTE-PDETKSTNFFGSKWNIKIGGLNGTTQDAT

* : *..**.: *.:*:* :. . :. :. : . : :.

23726RadD --------------AGTAKTAGKIVIEGNSSGSMGIYAKQGILELIKPHADSGETESS--

21_1ARadD GTKVEKAIGILQDHSSKTDTTAVIKIGEHSKGSIGVYGRQGILNITAPTAKDKILDSNNQ

CTI2RadD HGNTQTNPDSKNKVEQSIGLNFDFTIKNSGFKEREIKRYKVSLEADADSSTGIRVGTAKI

10953RadD HGNTQSNPNSASKVEQSIGLNFDFTVHASGLAQREIKKYKVSLEADADSSTGIRVGHAKI

. : : . . : : *: : .

23726RadD --------IELKAGSNNIGIFAKG-----ASTVNFNGITKITGGERQKIALATEGSTINL

21_1ARadD EKALTVSEIDLQGGADNIGIVASKDGTKPAAEVNVTGDVKISGGTGQKIAIAEKGGKINL

CTI2RadD NLTDADTFTQIKMNGTNNIGLLADGSSAELKYTNTRNALNLDGGSGNILFAAINSGKLNV

10953RadD NLTDADAFTQIKMNGTNNIGLLADGATSELKYTNTRNALNLDGGEGNILFASINSGKLNV

::: .. * . .* . :: ** : : : :...:*:

23726RadD KNTVTAGDKITSN-FVKDAVPLYATGTGSKINVVTPDSLKFYLGGNSTAAYAKDGGIINM

21_1ARadD KGNVMAGTNVN---FVKNAVPLYATGEHSTITVQNSNKFEFYLSGNSTAAYAKDKATINM

CTI2RadD ENKFELKTSGGNN-TTGAKFVSTYTKGAGSKVTFKKGISFNHTGDETVGMYATDGGNITV

10953RadD ENKFELKTSGGSDETTGAKFVSAYTKGSGSKVTFKKGISFKHTGNESIGMYATDSGDITV

:... . . . * .. . . . : ..:: . **.* . *.:

23726RadD N--------------RTNLPADPSIYIKG---------ENGKGIGLFAKDGGVINAQKHY

21_1ARadD N--------------RTTLPSEPTIHIKG---------ENGKGIGLFAKDGGVINAQKHY

CTI2RadD TNPTPVTLPVVTASSIIDNSTLPSIATSI---------TGSKSVGYYANNGGSIVNIGSS

10953RadD TNPTMVTLPTVTAASEIDNSTLPSVATTSPTISTKLSITGSKSVGYYANNGGTIVNIGSS

. .: *:: . ..*.:* :*::** *

23726RadD IKVENGSVALSSVGANATNKSNIDFTGGKLEYTGNGYAVYSDGTGTVNLSDAELNLYGSS

21_1ARadD IKVENGSTAISSIGAGSN----IDFTGGKLEYTGNGYAVYSDGTGKIDLSDAELNLHGSS

CTI2RadD TKITNGSALAYAKGANSK----ITISNSLLDYHGEGYSLYTENGGKIVADGSVLVLRGKA

10953RadD TKVTDGSALAYAKGANSK----ITISSSLLDYSGEGYSLYTENSGKIVADNSVLVLRGKA

*: :**. : **.:. * ::.. *:* *:**::*::. *.: ..: * * *.:

23726RadD TAFDVDFNATTLPTILNANTRIHANSDDVIAFNLKNASGLTTVGGIETSIKSKIETKLGL

21_1ARadD TAFDVDLGASTLPTKLNSGTKIKVHSDDVIAFNLKRATGLTTVGGIETSIKSKIETKLGL

CTI2RadD VGMKAKSVTSGDISFTNGKIVMMSNEAIPFVASDITGTVNTSDILGGIGLPSGITIAKGK

10953RadD VGMKANSVSSGDISFANGKIVMMSNDAIPFVASDITATVNASDIRTGLGLPSNIIIAKGK

..:... :: : *. : :. :. . .: :: .: * * *

23726RadD GSGSLNNLFTGSTSTKYKVAAVDGGEITVGNLDKSGTKDDTDQAKRDGYQYFNRFLAQRL

21_1ARadD GSGSLNNLFSGSTADRYKVAAVDGGEITVGNLDKSGTKDDTDQAKKDGYQYFNRFLAQRL

CTI2RadD EGGTVYNKYKIAAVDGMANLTIDQDLDKKYATDDN-NETSSDLSKKASYTLFRRYLIQRA

10953RadD EGGTVFNKYKIAAVDGMANLTIDYDLDKTYATDDN-NETSSDYSKKASYALFRRYLIQRA

.*:: * :. :: ::* . . *.. .: .:* :*: .* *.*:* **

23726RadD KATATGSTIKAVLNSNFANSNFNGQVVGFEMNSSKNATSVNETAINLVDSKIIADRTDAG

21_1ARadD KATANGSTIKAVLSSSFANDNFNGQVVGFEMNSSKNATNVDETAINLVNSKIMADRTDAG

CTI2RadD KVEASGKTIKAVLDSNDLTKLDATQVVGLEMSSSKNANNVNETAINLVNSKIIADRKDAG

10953RadD KVEANGKTIKAILNSTDLTKLDATQVVGLEMSSSKNANNVNETAINLVNSKIIADRKDGG

*. *.*.****:*.*. .. ****:**.*****..*:*******:***:***.*.*

23726RadD TGAIGAFINYGEVNIAATSKIEVEKEN---NVVNKQAVGVYAVNGSKVDNKGTIDVGGDQ

21_1ARadD AGAIGAFINYGLVNIDATSKIEVEKEN---NVVNKQAVGVYAVNGSKVDNKGTIDVGGDQ

CTI2RadD TGAVGAYINYGLVNIDATSKIEVEKDASAGNTANSGAVGVYAVNGSKVDNKGTIEAGGKE

10953RadD TGAVGAYINYGLVNIDATSKIEVEKDTSAGNTANSGAVGVYAVNGSKVDNKGNIEAGGTD

:**:**:**** *** *********: *..*. ****************.*:.** :

23726RadD SVGILGMAYREGSSNNPIVNEFGGKSG-EGTVNITNEKDIKMSGKDAIGIYAMNNNPDKT

21_1ARadD SVGILGMAYREDASHNPIVKEFGDKATNQGLVNITNEKDIKMSGKDAIGIYAMNNNTDTT

CTI2RadD SVGVLAMAYGE--SGGVTQKDQFGGKSGEGTFSVTNSGKITMSDDDAIGIYAKNNNTSAA

10953RadD SVGILALGYGE--SGGVAQKNQFGGKTGEGNITVTNSGSVTMANNDAIGIYVKNNNTTTV

***:*.:.* * * . :: . :* ..:**. .:.*:..******. ***. .

23726RadD VTSHLVINKGTVEVGDSAEKTAVGIYAKGVDVKPESGKIKIGKKAVGIYAEDSNVGEVNK

21_1ARadD VTSHLVTNKGTVEVGDSGEKTAVGIYAKGVNVKPESGKIKIGKKAVGIYAEDSQVGEANK

CTI2RadD SNDYKVTNTGTIEVKKSVSKTAIGIYADKSTVLPKDGTIKIGEKAVGIYAKDSIVGVAGN

10953RadD STNHKVTNSGTIEVKESASKTAIGIYADKSTVIPKDGTIKIGKKAVGIYADNSAIGSGTD

..: * *.**:** .* .***:****. * *:.*.****:*******.:* :* .

23726RadD DLGTIDFNGDDGVGIYLKGSGSNLLGNKVTLTQSKD--SKNKVGILADRGTSSIIKTEVA

21_1ARadD DLGTVDFNGDDGVGIYLKGSGSNLLGNKVTLTQSKD--SKNKVGILADRGTSSIIKTEVA

CTI2RadD NLGDIDFNGANGVGIYLKDN-STLLGNKVTLKQTATGTLAGKVGILADTLTNKTLNTEVV

10953RadD NLGKIDFAGESGVGIYLKGSSSTLTGSNVTLKQSTSGTFKGKVGILVARETASTITTEVK

:** :** * .*******.. *.* *.:***.*: .*****. * . :.***

23726RadD VG----ALNNVIAYYSKGNHEFNVQSNVTLNENSIGISGEGDLQYGDGTNP---YIMKLG

21_1ARadD VG----TLNNVIAYYSKGNHEFNVQSNVTLNENSIGISGEDDLLYGDGTNT---YTMKLG

CTI2RadD ADT---GVNDVIAYYSKGNGALTVEADISLNENSTGITG-GDTEDLVYSGS---KTMKLG

10953RadD TKDGSDTIDDVIAYYSKNNGTLTVQSSFELNKRSVGIYGAVNGSTPENLVYNGSKTMKLG

. :::*******.* :.*::.. **:.* ** * : ****

23726RadD KGSTGLFGTKNIVLKDKTNIELNGENSVGAYASGASGVITSEGKIKFLKEKSIGLYGANG

21_1ARadD KSSTGLFGTKKIGLKDKTNIELNGENSVGAYASGANGVITSEGKIKFLKENSIGLYGAKG

CTI2RadD KKSTGIYGQKNINFASGSNIELNGDKSVGIFAKGTSGIVSSNGNIKFTKENSIGLYGANG

10953RadD EKSTGIFGKGNISFASGSNIELNGDKSVGVFATESTGIINSEGNLKFSKEKSIGLYGLNG

: ***::* :* : . :******::*** :*. :.*::.*:*::** **:****** :*

23726RadD ATVKDKTT-MDFSNANAKNNIGVYLAGAKWDIDR--ALTFDSAHEKGNIYLFAQGGS---

21_1ARadD ATINDKTASMDFTNANAKNNIGVYLAGANWERDS--ALTFSSTHEKGNIYLFAQGGSEGA

CTI2RadD ATINDKIASMDFTNTNAKNNIGAYLAGAKWIDSRSGAYTFSPDHARNNIYLFAQGGNDGT

10953RadD ATINDKTASMDFSNANAKNNIGVYLAGAKWNDNRTAAYTFNSDHSKNNIYLFAQGSRING

**::** : ***:*:*******.*****:* . * **.. * :.********.

23726RadD -----TATLKNGFDITPLTAPTGNNRTIGMYLDTAVKGGATTADNTVDMSDGNAKISVTK

21_1ARadD TDKGNKITLKNIFNVSPSNDPTGNEKTIGMYLDTAVKGKSTYVDNTVDMSDGNAKVSVTK

CTI2RadD TDLGSTATLNNEFKVNPSGSATSTAKAIGMYFNTAVKDKTTFVDNTLNMTASNAKISVIN

10953RadD TDTGSTVTLKNDFQVAPGGSASPAEKTIGMYFDTTVKGG-TFAENKVDMTNANAKVSVTN

. **:* *.: * .: ::****::*:**. * .:*.::*: .***:** :

23726RadD KAIGIYAKNVDNS--KNNIINKIKVLSDGQGTVGVFTDGNLKLSGNGGLIEAQNAGIGLY

21_1ARadD KAIGIYAKNADNS--KNNIINTLKVSSAGQGTVGVFTDGNLKLSGNGGLIEAKNSGIGLY

CTI2RadD SGIGVYAKNTTGSG-KNNIINKINVSSSGSGSVGVFTDGDLKLSGATGLIEAKNSGIGLY

10953RadD KGIGLYTKAEATSSGKNNIIEKIKVSSTGKDSVGVYNKGNLKLSGTDGLIEAKTNGIGIY

..**:*:* * *****:.::* * *..:***:..*:***** *****:. ***:*

23726RadD GNKGTVTVEGTHKVEVTSAGTGMYLTKGSHLSGGKLELENKTAGTSAAGIYYEGTN--NE

21_1ARadD GNKGTVTVEGTHKVEVSSAGTGMYLTKGSHLSGGKLELENKTAGTSAAGIYYEGTG--NE

CTI2RadD GNSGKVTVDGKHKVEVTSAGTGMYMTNGSYLEGGELELKNNTAGTAAAGIYYTKGNNSNE

10953RadD GVAGKITVENTHKVEVTAAGTGMYLTGNSYLDGGKLKLENKTVGTSAAGIYYKKGTSSDE

* *.:**:..*****::******:* .*:*.**:*:*:*:*.**:****** :*

23726RadD VDHNTDIEVTAGENLLAIYANG-LKLNNNKEILIKKGKNNVAAYITGNSTFKNKGKIQLG

21_1ARadD VNHDTDIVVTAGENLLALYANG-LKLNNNKEIIIKKGKNNVAVYITGNSTFRNKGKIQLG

CTI2RadD VTHNTDLKVNSENNLLALYADGGIKLNNAKTIEIGDGENNVGAFVTGNSIFKNKGKISLT

10953RadD VTHNTDLEVVSGSNLLALYADGGIKLNNAKAINITDGANNVGVFVTGGSTFKNTGSIVSG

* *:**: * : .****:**:* :**** * * * .* ***..::**.* *:*.*.*

23726RadD HPTQN-DFESGIGIYVVDGEAINESGKTIDIYDFENTASGGSLSVGMLAKAGAGKTAKVT

21_1ARadD ESGNNGDFKSGIGVYVVDGEAINETGKTIDIHDFNDEAG---LSVGMLANAASGKTAKVT

CTI2RadD GSIKN-----AIGVYVEDGEATNEAGKDIEILDLNTSGTG-TPSIGMTAKAASGKTAKIT

10953RadD SASNS-SIKNAIGVYVEDGEAKNESGKSIEVYDLNTSSGG-TLSIGMIAKAATGKTAKVI

. :. .**:** **** **:** *:: *:: . *:** *:*.:*****:

23726RadD NKGTINANGEVIGMVVEDNSEGLNDTGAEIVAKDKEDINAKAIGAYVKGANAKFENKGKI

21_1ARadD NKGTINANGEVIGMVVEDNSEGINDTGAEIVAKNEE--PLKAIGAYVNGANAKFENKGKI

CTI2RadD NKGTINAKGEAIGMNVEDNSEGENTGTIESKNWSLAGNTFKSIGAYINGGNAKFTNSGTI

10953RadD NAGTIKVLGEAIGMNIENNSEGANSGTITATDKEISGTNYKSIGAYINGANAKFTNTGTI

* ***:. **.*** :*:**** * . *:****::*.**** *.*.*

23726RadD SAENIALALQGTGANKILNSGTLNLTKTGAIGVYAKDSVVDFNIAPTVAG-ANKTVALYA

21_1ARadD SAENIALVLQGTKEGNIKNTGTLNLTKTGAVGVYAKDSVVDFNIAPTVAG-ADKTVALYA

CTI2RadD SAENIGLALKDTAANKILNSGNLKLTKSEAVGVYANNSIVDFNIAPTVASGIDKTVALYA

10953RadD STENIGLALKDTTANKILNSGTLKLTKTGAVGVYANNSIVDFNITPTVASTVDKTVALYA

*:***.*.*:.* .:* *:*.*:***: *:****::*:*****:****. :*******

23726RadD SGTTKIKSQITSASGKAHIGVYAEGDAEFLSGSKVTVGNGDGND----YGIGVYTKSGYN

21_1ARadD SGTTKIKSQITSATGKAHIGVYAEGNAEFLSGSKVTVGNGSGND----YGIGVYTKSGYN

CTI2RadD SGTTKIKSQITSASGKAHIGVYAEGDAEFLSGSKVTVGNGDGND----YGIGVYTKSGYN

10953RadD TGTTKIKGQITTAAGKSHVGVYAEGNAEFQSGSKVTVGDGIEDSGTTYYGIGIYTKSGYN

:******.***:*:**:*:******:*** ********:* :. ****:*******

23726RadD KTVNTDIQLGGEKTIGFYLGATGGTGSTVTHTGTIDVGSGIGTYIPEHSKFIAQNTTFNV

21_1ARadD KTVNTDIQLGGEKTIGFYLGATGGSGSTVTHNGTINVGSGIGAYIPEHSKFIAQNTTFNV

CTI2RadD KTVNTDIQLGGEKTIGFYLGATGGTGSTVTHTGTIDVGSGIGTYIPEHSKFIAQNTTFNV

10953RadD KAANVNLKLNGSKAIGFYLGAAGTTGSTVTHTGTIDVGSGIGTFIPKYSKFVAQNTTFNV

*:.*.:::*.*.*:*******:* :******.***:******::**::***:********

23726RadD GDNGTAVYLKGGEVDLGKTGTANINFNGTNGRAIYQDGGTITTGTGLHIQGSGSFLTLKN

21_1ARadD GDKGTAVYLKGGEVDLGKTGTANINFNGTNGRAIYQDGGTITTGTGLHITGSGSFLTLKN

CTI2RadD GDNGTAVYLKGGEVDLGKTGTANINFNGTNGRAIYQDGGTITTGTGLHIQGSGSFLTLKN

10953RadD GNGGTAVYLKGGEADLGSTGTANINFTGS-GRAVYQDGGTLTTGAGLHITGTGSFLTLKN

*: **********.***.********.*: ***:******:***:**** *:********

23726RadD ANSSINSLVEVGASGIGINGIYDMAGKDYTLTLESPNGHIKLGGNKATGIAAVAKSTVGP

21_1ARadD ANSSINSIVEVGANGIGINGIYDKS-GTYKLTLESPNGHIKLGGDKGTGIAAIAKNTAG-

CTI2RadD ANSSINSLVEVGASGIGINGIYDMAGKDYTLTLESPNGHIKLGGNKATGIAAVAKSTVGP

10953RadD ANSIINSIVNVGADGIGINGIYDSNAQDYTLKLDSPTGHIKLSGNKATGIAAVAKNTVAP

*** ***:*:***.********* *.*.*:**.*****.*:*.*****:**.*..

23726RadD NKVNVINKGTIETTSGEKTTGIYGKGANIENATGAKINIGAKGVGIYTTNDNS-----LE

21_1ARadD LKVDIINKGIIETTSGEKTTGIYGKGANIENATGAKINIGAKGVGIYTTNDNS-----LE

CTI2RadD NKVNVINKGTIETTSGEKTTGIYGKGANIENATGAKINIGAKGVGIYTTNDNS-----LE

10953RadD KKVDIINRGTIETISGSETTGIYGKGANIENATGAKINIGAKGVGIYTTNYYDNSTLSLT

**::**:* *** **.:******************************** . *

23726RadD NTTLNNAGEINLTGDEATGLVAVKAKTNQDFIVGKISGTKDKLVGAYFKDSQAVTKVKDF

21_1ARadD DTTLNNAGEINLIGDEAKGIVAIKSNTNQDFIVGKITGTKDKLVGAYFKDSQAVTKVKDF

CTI2RadD NTTLNNAGEINLTGDEATGLVAVKAKTNQDFIVGKISGTKDKLVGAYFKDSQVVTKVKDF

10953RadD NTTLKNNGEINLTGDEATGIVAVKAHTTQDFIGGKITGTKDKLVGMFFDNSAAQTKVKDF

:***:* ***** ****.*:**:*::*.**** ***:******** :*.:* . ******

23726RadD NISLGTNAKGLVFDGGKDFTVTSSSTNKVKIG-NTTGNSRGIGIAALGVNGNISKTDVVV

21_1ARadD NISLGTNAKGLVFNEGKDFTITSSSTNKVTIG-ATTGNSRGIGIAALGVNGNISKTDVVV

CTI2RadD NISLGTNAKGLVFNGGKDFTITSSSTNKVTIG-ATTGNSRGIGIAALGVNGNISKTDVVV

10953RadD NISLGTNAKGLIFKNGQDFTITSSSTNKVTIGNTTNVASHGIGISALGVNGTVSNTEVKV

***********:*. *:***:********.** *. *:****:******.:*:*:* *

23726RadD GKGSLGLYAKDKKLTFDLASGKLESSDANRSSILAYADGNTSEVALNGGGTLKVGANGIA

21_1ARadD GKGSLGLYVKNKKLTFDLATGKLESSDASRSSILAYADGNNSEVALNGGGTLKVGANGIA

CTI2RadD GKGSLGLYAKDKKLTFDLASGKLESSDANRSSILAYADGNTSEVALNGGGTLKVGANGIA

10953RadD GKNSLGLYAKDKKLTFDLATGKLESSDSGRSSILAYADGNNSEVALNGGGTLKVGANGIA

**.*****.*:********:*******:.***********.*******************

23726RadD LGTKGGKVSANATTTVEVDGVKGLGAYVENGGSIDNNFDIKVKSAEGIGMYAKGGALTSV

21_1ARadD LGTKGGKITANATTTVEVDGVKGLGAYVENGGSISNNFDIKVKSAEGIGMYAKGGALASV

CTI2RadD LGTKGGKVSANATTTVEVDGVKGLGAYVENGGSIDNNFDIKVKSAEGIGMYAKGGALTSV

10953RadD LGTKGGKITANVTTNVEVDGVKGLGAYVENGGSISNNFDIKVKSAEGIGMYAKGGALTSV

*******::**.**.*******************.**********************:**

23726RadD AKVSEIKGNKSIGYVFENITSAITMPNSVQLTDINATGQVGVVAQGTGNGLTVAGVSVVG

21_1ARadD AKVSELKGNKSIGYVFENITNAINMPNSVQLTDTNATGQVGVAVKGTGAGLTVAGVYVVG

CTI2RadD AKVSEIKGNKSIGYVFENITSAITMPNSVQLTDTNATGQVGVVAQGTGNGLTVAGVSVVG

10953RadD AKVSELKGNKSIGYVFENITNAITMPNSVQLTDTNATGQVGVVAQGTGNGLTVTGISVVG

*****:**************.**.********* ********..:*** ****:*: ***

23726RadD SGNTGVYSSTGKAVINNGTLTVGDSTGKSSIGIYSKGGAVTSTGSATIGKNSIAIYGKDT

21_1ARadD SKNIGIYNETTGAVTNNGALNVADSTGDSSIGIYSQGGTVTSTGNATIGKNSIAIYGKNT

CTI2RadD SGNTGVYSSTGKAVINNGTLTVGDSTGKSSIGIYSKGGAVTSTGSATIGKNSIAIYGKDT

10953RadD SENTGVYSSTGKAVVNNGTLTVGDSTGKSSIGIYSKEGAVTSIGNATIGKNSIAIYGKDT

* * *:*..* ** ***:*.*.****.*******: *:*** *.*************:*

23726RadD AATLNGNLTIGEKGIGLYVDNTATSKGDTAVNGNITVGANGAIGIQTTNSKVNLTGDLSV

21_1ARadD AATLNGNLNIGEKGVGLYVDNTATSKGDTAVNGNITVGANGAIGIQTTNSKVNLTGDLSV

CTI2RadD AATLNGNLTIGEKGIGLYVDNTATSKGDTAVNGNITVGANGAIGIQTTNSKVNLTGDLSV

10953RadD AATLNGNLNIGEKGIGLYVDNTATSKGDTAVNGNITVGANGAIGIQTTNSKVNLTGDLSV

********.*****:*********************************************

23726RadD ASGDSKGIFSMGAGNVETTGNITVGSNSVGIYKNGSGEVKTAAG---KTLTVADSAYGIF

21_1ARadD ASGDSKGIFSMGAGNVETTGNINVGNNSVGIYKNGSGEIKTALGSIGKTLTVADSGYGVF

CTI2RadD ASGDSKGIFSMGAGNVETTGNITVGSNSVGIYKNGSGEVKTAAG---KTLTVADSAYGIF

10953RadD ASGDSKGIFSMGAGNLETTGNINVGNNSVGIYKNGSGEIKTALGSIGKTLTVADSGYGVF

***************:******.**.************:*** * ********.**:*

23726RadD SKGAKLINNMNVTVGVDAIGAYVDGNDLTSTGTVTVGDKGVGLFVKGTGKTLTSTGNITV

21_1ARadD SKGAKLINNMNVTVGVDAIGAYVDGNDLTSTGTVTVADKGVGLLVKGLGKTLTSTGNITV

CTI2RadD SKGAKLINNMNVTVGVDAIGAYVDGNDLTSTGTVTVGNKGVGLLVKGTGKTLTSTGNITV

10953RadD SKGAKLINNMNVTVGVDAIGAYVDGNDLTSTGTVTVADKGVGLLVKGTGKTLTSTGNITV

************************************.:*****:*** ************

23726RadD GSNNSVGLYAGDNANIAQSGNITVANNNGIGVYSKGNGNVSTIGAITVGKDSIGVYKDGK

21_1ARadD GSNNSVGLYAGDNANIAQSGNITVANNNGIGVYSKGSGNISTVGAMTVGKDSIGVYKDGK

CTI2RadD GSNNSVGLYAGDNANIAQSGNITVANNNGIGVYSKGSGNISTVGAMTVGKDSIGVYKDGK

10953RadD GSNNSVGLYAGDNANISQSGNITVADNNGIGVYSKGSGNISTVGAMTVGKDSIGVYKDGK

****************:********:**********.**:**:**:**************

23726RadD GTMNINASSPIQTMTIAEKGYGLYYKGNSRADSIINSNMNMTLGKEAVGIYAKNTTVNHV

21_1ARadD GTMNINASSPIQTMTIAEKGYGLYYKGNSRADSIINSNMNMTLGKEAVGIYAKNTTVNHT

CTI2RadD GTMNINASSPIQTMTIAEKGYGLYYKGNSRADSIINSNMNMTLGKEAVGIYAKNTTVNHT

10953RadD GTMNINASSPIQTMTIAEKGYGLYYKGNSRADSIINSNMNMTLGKEAVGIYAKNTTVNHT

***********************************************************.

23726RadD GDITVGETNIGSSGFTTPSDNKNSIGIFGDNSNINFKGNMLVDKPLSVGIYGSNGGSITV

21_1ARadD GDITVGETTIGSSGFTTPSDNKNSIGIFGDNSNINFKGNMLVDKPLSVGIYGANGGSITV

CTI2RadD GDITVGETTIGSSGFTTPSDNKNSIGIFGDNSNINFKGNMLVDKPLSVGIYGANGGSITV

10953RadD GDITVGETTIGSSGFTTPSDNKNSIGIFGDNSNINFKGNMLVDKPLSVGIYGANGGSITV

********.*******************************************:*******

23726RadD KSGSTITVKNGATGIMTGSKVESITLESGSTLNVDGKVDTSVYTNATKSNISFGIAAYSG

21_1ARadD KSGSTITVKNGATGIMTGSKVENITLESGSTLNVDGKVDTSVYTNATKSNVSFGIAAYSG

CTI2RadD QSGSTITVKNGATGIMTGSKVENITLESGSTLNVDGKVDTSVYTNATKSNVSFGIAAYSG

10953RadD KSGSTITVKNGATGIMTGSKVENITLESGSTLNVDGKVDTNVYTNATKSNVSFGIAAYSG

:*********************.*****************.*********:*********

23726RadD AINNQGTINVTNGATGIYLAGTASLVN--QGTITVDAISKQIGRPDTKASAELGGIKVTD

21_1ARadD LIDNQGTINVKNGATGIYLAGTASLKNGATGTINIDATSKSTAKPDTKASAELGGIKVTD

CTI2RadD LIDNQGTINVKNGATGIYLAGTASLKNGATGTINIDATSKSTAKPDTKASAELGGIKVTD

10953RadD LIDNQGTINVTNGATGIYLAGTASLKNGATGTINIDATSKSTAKPDTKASAELGGIKVTD

*:*******.************** * ***.:** **. .:****************
